# Comparative proteomics of wild-type and nef-deleted HIV-1 particles

**DOI:** 10.1186/1742-4690-8-S2-P3

**Published:** 2011-10-03

**Authors:** Christelle Brégnard, Olivier Danos, Stéphane Basmacioqullari

**Affiliations:** 1 Hôpital Necker-Enfants Malades, Université Paris Descartes, Paris, 75743 Cedex 15, France; 2 INSERM U845, Paris, 75730 Cedex 15, France; 3 Cancer Institute, University College London, London, UK

## Background

Human and Simian Immunodeficiency viruses acquired auxiliary genes that allowed a better adaptation of viruses to their hosts. Among these genes, *nef* was shown to play an important role in the onset of immunodeficiency syndromes. Humans infected with viruses harboring major deletions in *nef* resulting in no Nef expression (Δ*nef*) remain symptom-free for a significantly longer period of time than individuals infected with wild type (WT) viruses. Single round infection assay also reveal that WT viruses are 5-10 fold more infectious than their *Δnef* counterparts. This phenotype relies on a function of Nef that takes place during viral particles biogenesis. A proteomic analysis of WT and *Δnef* viruses was conducted to identify differences responsible for the higher infectivity of WT viruses.

## Materials and methods

Virions were harvested from cell culture supernatant of 293T cells transfected either with pNL4-3 or pNL4-3 *Xho* in which the Nef ORF has been disrupted. Supernatants were then ultracentrifuged over two 20 and 60% sucrose cushions. Viral like particles were harvested at the 60-20% interface, diluted in PBS and ultracentrifuged again. Pelleted material was then subjected to Differential Gel Electrophoresis (DIGE) or Isobaric Tagging for Relative and Absolute Quantification (ITRAQ). Proteins enriched either in WT or Δ*nef* virions were selected for further characterization in order to investigate their role in virus infectivity. Candidate proteins were over expressed in WT or *Δnef* single round infection competent virus-producing cells, alternatively, siRNA was used to silence candidate protein expression.

## Results

DIGE and ITRAQ revealed that Nef regulates the incorporation/ exclusion of cellular proteins into/from virions. Both methods showed that Glucosidase II and ERM proteins are enriched in WT and *Δnef* virions, respectively. In addition, ITRAQ which is more sensitive that DIGE, pointed out other differences, among which CD81, ALG-2 and EHD4, all enriched in *Δnef* virions, were selected for further characterization, based on their involvement in mechanisms that potentially affect virus biogenesis. Over expression or silencing CD81 in virion-producing cells decreased or increased virus infectivity, respectively. In addition, CD81 decreased virus release. Both effects were observed in HXBc2 env pseudotyped virions, not on VSV-G pseudotyped virions. Silencing Ezrin also increased HXBc2 pseudotyped virus infectivity. Although Ezrin over expression affected neither virus infectivity nor release, over expression of the FERM domain of Ezrin significantly decreased virus release. Glucosidase II, ALG-2 and EHD4 are presently under investigation.

## Conclusions

The presence of a different set of cellular proteins in WT and *Δnef* HIV-1 particles might affect virus infectivity. We clearly demonstrated that Ezrin and CD81 are less abundant in WT virions than in *Δnef* virions, which correlates with a higher infectivity of WT virions. This could be recapitulated by artificially manipulating the expression level of both proteins in virion producing cells. Interestingly, Nef potently increased the infectivity of virions produced in cells depleted from CD81, or, to lower extent, Ezrin. This suggests a partial overlap between the ability of Nef to exclude CD81/Ezrin from virions in the course of their biogenesis, and to increase virus infectivity.

